# Forms and functions of bridging factors: specifying the dynamic links between outer and inner contexts during implementation and sustainment

**DOI:** 10.1186/s13012-021-01099-y

**Published:** 2021-04-01

**Authors:** Rebecca Lengnick-Hall, Nicole A. Stadnick, Kelsey S. Dickson, Joanna C. Moullin, Gregory A. Aarons

**Affiliations:** 1 The Brown School, Washington University in St. Louis, St. Louis, USA; 2 Department of Psychiatry, University of California-San Diego, La Jolla, USA; 3 UC San Diego Altman Clinical and Translational Research Institute Dissemination and Implementation Science Center, La Jolla, CA USA; 4 Child and Adolescent Services Research Center, San Diego, CA USA; 5 College of Education, San Diego State University, San Diego, CA USA; 6 Curtin Medical School, Curtin University, Perth, Western Australia

**Keywords:** Bridging factors, EPIS framework, Outer and inner context, Forms, Functions, Implementation, sustainment, barriers and enablers, evidence-based practice

## Abstract

**Background:**

Bridging factors are relational ties, formal arrangements, and processes that connect outer system and inner organizational contexts. They may be critical drivers of evidence-based practice (EBP) implementation and sustainment. Yet, the complex interplay between outer and inner contexts is often not considered. Bridging factors were recently defined in the updated Exploration, Preparation, Implementation, Sustainment (EPIS) framework. Further identification and specification of this construct will advance implementation models, measures, and methods. Our goal is to advance bridging factor research by identifying relevant dimensions and exemplifying these dimensions through illustrative case studies.

**Methods:**

We used a multiple case study design. Each case (*n* = 10) represented different contexts, EBPs, and bridging factor types. Inclusion criteria were the presence of clearly distinguishable outer and inner contexts, identifiable bridging factor, sufficient information to describe how the bridging factor affected implementation, and variation from other cases. We used an iterative qualitative inquiry process to develop and refine a list of dimensions. Case data were entered into a matrix. Dimensions comprised the rows and case details comprised the columns. After a review of all cases, we collectively considered and independently coded each dimension as function or form.

**Results:**

We drew upon the concepts of functions and forms, a distinction originally proposed in the complex health intervention literature. Function dimensions help define the bridging factor and illustrate its purpose as it relates to EBP implementation. Form dimensions describe the specific structures and activities that illustrate why and how the bridging factor has been customized to a local implementation experience. Function dimensions can help researchers and practitioners identify the presence and purpose of bridging factors, whereas form dimensions can help us understand how the bridging factor may be designed or modified to support EBP implementation in a specific context. We propose five function and three form bridging factor dimensions.

**Conclusions:**

Bridging factors are described in many implementation models and studies, but without explicit reference or investigation. Bridging factors are an understudied and critical construct that requires further attention to facilitate implementation research and practice. We present specific recommendations for a bridging factors research agenda.

**Supplementary Information:**

The online version contains supplementary material available at 10.1186/s13012-021-01099-y.

Contributions to the literature

- This work advances the conceptual basis and study of bridging factors and is the first manuscript to specify and define bridging factor dimensions for implementation research.
- Through our novel application of the functions and forms framework, we propose a core set of dimensions that lays the groundwork for bridging factor reporting and measurement.
- Enhanced operationalization of bridging factors will help researchers and practitioners proactively leverage strategies that can strengthen linkages across outer and inner contexts to improve implementation outcomes and public health impact.

## Background

The implementation and sustainment of evidence-based practices (EBPs) requires simultaneous and continued coordination, support, and engagement of actors across outer and inner contexts [1–4]. Bridging factors span the outer and inner contexts and are crucial for this to occur. Bridging factors are defined as “factors that cross or link the outer system and inner organizational context” [5]. To date, there has been a heavy focus on inner context implementation work [5–7]. Research on the outer context has focused on how to affect policy within the outer context (e.g., through  targeted packaging and dissemination of research evidence) in ways that can be used at different jurisdictional levels, including whole countries [8, 9]. Although there have been calls for further efforts to transfer knowledge to outer context policymakers and system leaders [10, 11], this knowledge transfer has lagged in implementation science. Furthermore, less attention has been paid on how to leverage bridging factors between the outer and inner contexts to support EBP implementation and sustainment.

Bridging factors are the connective tissue between system parts and may be broadly organized as relational ties, formal arrangements, and processes [12]. Bridging factors may be relational ties, such as a partnership between a government agency and community-based organizations, or an EBP intermediary that facilitates intervention adaptation and training with system and organizational leaders [5, 12]. They may also be formal arrangements, including contracts between public sector agencies and local nonprofits, or a policy that provides a fiscal incentive for EBP use and facilitates EBP integration [12]. Other bridging factors may be process-oriented, such as data sharing procedures between state and local entities or accreditation, which links program developers with implementing organizations [12].

### The need for a bridging factors research agenda to advance implementation research and practice

We assert that identifying and leveraging bridging factors is an urgent priority for implementation research. First, understanding the interconnections between outer and inner contexts may be critical for EBP implementation and sustainment. This need is espoused by multiple implementation framework developers, but is often not addressed in implementation research. For example, the Dynamic Sustainability Framework states that further specification of the levels within a system and the interrelationships between them is an important area for future research [13]. The Integrated Sustainability Framework also notes that “dynamic interactions between outer contextual factors, inner contextual or organizational factors, processes, intervention characteristics, and implementer characteristics influence sustainability” [14]. The bridging factors construct can help us outline how specific relational structures, formal arrangements, and processes that connect outer and inner contexts support or hinder EBP sustainment.

Bridging factors research is also an urgent priority because it can advance the range of existing conceptual frameworks that acknowledge both outer and inner contexts for EBP implementation. Recent work identifies bridging factors in the Exploration-Preparation-Implementation-Sustainment (EPIS) framework and demonstrates a methodological approach for studying bridging factors using contracting arrangements as an example [5, 12]. However, there is variability in the acknowledgement of the bridging factors construct within other commonly used implementation frameworks. For example, the Consolidated Framework for Implementation Research (CFIR), Reach Effectiveness-Adoption Implementation Maintenance (RE-AIM), and Interactive Systems Framework (ISF) allude to the reciprocal effects, processes, or influences between outer system and inner organizational implementation actors [15–17]. The CFIR is inclusive of the outer and inner context stakeholders who may take part in activities in the Process domain [15]. The ISF explicitly includes the reciprocal effects between the “delivery system,” “support system,” and “synthesis and translation system,” all of which span the outer policy and inner organizational contexts [17]. Explicitly including bridging factors as a way to understand the relationships between outer and inner contexts can enhance our use of existing implementation frameworks.

Bridging factors are distinct from implementation strategies. However, they can enhance two streams of implementation strategy research and practice: (1) multifaceted, large-scale strategies (e.g., those that involve entities within an entire county, province, prefecture, or state), and (2) discrete, inner context-focused strategies. Multifaceted, large-scale implementation strategies that require coordination among outer and inner actors are understudied compared to other strategy types [10, 18–21]. Examples in the Expert Recommendations for Implementing Change project include accessing new funding, altering incentives for implementation or creating disincentives (de-implementation) structures, modifying payments or fees, building a coalition or community-academic partnership, and staging implementation scale-up [22]. Bridging factors may be planned implementation strategies or an unplanned contextual feature that activates or impedes the use of a multifaceted, large-scale strategy. For example, a community-academic partnership may consist of outer context actors (the academic institution and service system leaders) and inner context actors (in local organizations). The partnership may be created to intentionally support the implementation of a new EBP. However, this partnership could also be an existing contextual feature that helps to activate a different multifaceted implementation strategy (e.g., staging EBP scale-up or designing new incentive or disincentive structures). This example shows how bridging factors can be applied to existing implementation research topics including community-academic partnerships (e.g., [23–25]), learning collaboratives and learning communities (e.g., [26–28]), and inter-organizational collaboration [4, 29].

Identification of bridging factors can also enhance research on discrete, inner context-focused implementation strategies. Although much progress has been made in specifying and reporting discrete implementation strategies, we often do not account for the outer-inner context dynamics that these strategies are used within. In fact, information about context is noticeably absent in published reporting guidelines [27, 30]. This tension is exemplified in technology-enabled services implementation (i.e., online or mobile platforms to deliver healthcare) wherein the ability to execute common inner context-focused implementation strategies may be supported or constrained by the relationship that inner context actors have with the broader service environment [31].

In another example of a recent organizationally-focused implementation trial, a statewide policy initiative distracted parties (across all levels of the organization, including executives, supervisors, and providers) from the specific EBP implementation goal, as they were required to meet new documentation and process obligations to maintain their organizational funding [32, 33]. Without specifying this bridging factor (the statewide policy initiative), it may appear that the implementation strategy itself was less effective. Specifying and reporting bridging factors can help implementers adapt inner context-focused strategies to fit outer context characteristics and disruptions.

Further, this example illustrates how the success of the same strategy in different settings may be more fully explained by bridging factors. In the context of implementation research, mechanisms are defined as “a process or event through which an implementation strategy operates to affect desired implementation outcomes” [34]. Mechanisms are multilevel and can be activated by implementation strategies at intrapersonal, organizational, community, and macro policy levels of analysis [34, 35]. While a bridging factor in and of itself is not a mechanism, explicating relevant bridging factors can shed light onto multilevel mechanisms that explain implementation strategy effectiveness. The bridging factors construct draws our attention not only to the multilevel nature of strategies and their mechanisms, but also to the bi-directional (e.g., top down and bottom up) processes that may explain how a strategy works.

### Integrating bridging factors with existing organizational theories and concepts

We acknowledge that organizations operate within the external environment and this is the basis of longstanding theories that fall within a broader open systems perspective. Open systems theorists treat organizations as permeable. The external environment “shape[s], support[s], and infiltrate[s] organizations” and organizations exchange resources, information, and personnel with entities outside the boundaries of the inner context [36]. Complexity theory, for example, centers on how systems consist of multiple components that relate toand interact with one another in dynamic ways [37, 38]. Transaction cost economics, institutional, contingency, and resource dependence theories can also help to frame research questions about how the outer context influences the inner organizational context over the course of implementation [39–43].

Of note, bridging factors are alluded to in resource dependence theory and are described as an organization’s “efforts to control or in some manner coordinate one’s actions with those of formally independent entities” [36]. According to this theory, specific bridging tactics include forming alliances or merging with other organizations, and each tactic represents a way that an organization can manage interdependence with the broader environment [36]. These bridging tactics may be operationalized within the construct of bridging factors by looking at specific formal arrangements (e.g., contracts between organizations) or processes (e.g., data sharing or staff integration processes when a merger takes place) that influence EBP implementation.

There is also the concept of bridging organizations, defined as independent organizations whose role is to link center (high status, high power) and border (low status, low power) organizations [44]. Bridging organizations bring together diverse stakeholders to facilitate inter-organizational collaboration and coordination [45]. They help to build trust and resolve conflict, and provide an opportunity for sensemaking and learning across organizations [45]. They can also help disseminate shared visions and new innovations [46]. EBP intermediary/purveyor organizations are an example of bridging organizations that connect intervention developers with implementing organizations [47]. They build agency and system capacity through consultation, technical assistance, outcome evaluation, and training in the intervention [47]. Bridging organizations may be in the relational tie category of bridging factors.

Finally, there are similar individual and team level concepts in the social network literature, for example, bridges and brokers, which fall within the broader concept of boundary spanners [48–53]. Boundary spanners close structural holes between networks and serve many of the same functions as bridging organizations, e.g., building trust, resolving conflict, and acting as a conduit for resource and information exchange [49, 54, 55]. Individuals or teams who serve a boundary spanning role between different outer and inner context social networks may also be in the the relational tie category of bridging factors. Bridging factors research can supplement existing theories by identifying concrete and malleable bi-directional linkages, and articulating the dynamic interplay that occurs between organizations and the outer context as an EBP becomes embedded in an implementation setting.

### Summary of our argument

We argue that enhanced identification, operationalization, and understanding of bridging factors will allow researchers and practitioners to proactively investigate and leverage them during implementation efforts. This can support the development of strategies that support implementation processes --from adoption to sustainment-- and improve the public health impact of EBPs. The goals of this paper are to (1) raise awareness about the importance of specifying and reporting bridging factors, (2) advance the bridging factors construct through a presentation of dimensions and illustrative case studies, and (3) discuss recommendations for bridging factors research.

## Methods

### Case selection

We used a multiple case study design [56] and iteratively reviewed cases to inform the development and refinement of a list of dimensions of bridging factors. We assessed the unique features of each case and patterns across the cases [56]. Cases were purposefully sampled to ensure diversity across EBP, context, and bridging factor type. To ensure breadth of examples, we drew upon cases from the authors’ research experiences and from discussions with colleagues who conduct multilevel implementation research and engage in implementation practice. During these discussions, we sought to gather enough detail to understand what the bridging factor was and identify specific ways that it affected implementation processes in both the outer and inner contexts. Inclusion criteria for the cases were (1) the presence of clearly distinguishable outer and inner contexts, (2) an identifiable bridging factor, (3) sufficient information to describe how the bridging factor affected implementation, and (4) variation from other cases.

### Code development and application

Each member of the research team independently generated a list of potentially relevant bridging factor dimensions. Following independent list development, we convened for six consensus-building meetings to create and refine a dimension list, discuss each dimension’s purpose (function or form), and compare our conceptualization of the dimension with material from the cases [57]. During the meetings, we developed and used a matrix whereby the list of dimensions comprised the rows and examples from the cases comprised the columns [58]. Based on our discussion of the cases and our collective reflection about the type of information gleaned for each of the dimensions, we (RL, NS, KD, and JM) independently coded each dimension as one that describes the function or the form of the bridging factor (described below). Given the complexity of the form dimensions, we anchored sub-categories within broader categories to organize this information. We discussed each discrepancy and GA acted as a tiebreaker because he is the most senior researcher and experienced in cross-context implementation research.

### Sample

We reviewed 10 cases representing three types of bridging factors: relational ties (*n* = 5), formal arrangements (*n* = 3), and processes (*n* = 2). Four cases were excluded from our final sample because the EBP was already represented in two other SafeCare-focused cases (*n* = 1), detailed information was not publicly available (*n* = 1), outer and inner context boundaries were not sufficiently clear (*n* = 1), and the information provided was not distinct enough from other relational tie examples (*n* = 1). The outer contexts in our sample were a public sector child welfare system, public sector mental health system, local government in low- and middle-income country (LMIC) setting, substance use treatment organizations in the community, the State, local and state health departments, program developers, and a university medical center. The inner contexts were organizations delivering the EBP(s), churches, local child welfare agencies, public sector mental health systems, and a state-run prison. The cases illustrated bridging factors across very different context and service settings. Case information is summarized in Table 1 and full case study reports are presented in the Additional file 1.

**Table 1:** Summary of case studies

Case number and title		1. Contracting arrangements	2. Policy-driven fiscal incentive	3. Community-academic partnerships in a LMIC	4. START model partnerships	5. Earmarked taxes	6. State-wide interagency collaboration	7. Data sharing process	8. Partnership between state and local child welfare agencies	9. Site-level accreditation process	10. Individual as a bridging factor
**Study details**											
**Intervention "The thing"**		SafeCare^®^	Multiple EBPs	Healthy Beginnings	START model	Not EBP specific	EBPs for autism spectrum disorder	Evidence-based HIV interventions	R^3^ supervision focused implementation approach	SafeCare^®^	Programming for incarcerated pregnant and postpartum women
**Bridging factor**		Contracting arrangements between public sector child welfare systems and organizations delivering the EBP	Policy-driven fiscal incentive that connected a public sector mental health system and organizations delivering the EBPs	Partnership between the local government in LMIC setting and churches	Partnership between substance use treatment organizations and local child welfare agencies	Earmarked taxes that connected states with public sector mental health systems and organizations delivering the EBPs	Interagency collaboration among a state and organizations delivering the EBPs	Data sharing process that connected local and state health departments with organizations delivering the EBPs	Partnership between state and local child welfare agencies	Site-level accreditation process that connected program developers with organizations delivering the EBP	An individual who connected a university medical center with a state-run prison
**Function dimensions**											
**1**	**Type**	Formal arrangement	Formal arrangement	Relational tie	Relational tie	Formal arrangement	Relational tie	Process	Relational tie	Process	Relational tie
**2**	**Outer context**	Public sector child welfare system	Public sector mental health system	Local government in LMIC setting	Substance use treatment organizations in the community	State(legislators and voting constituents)	State (policymakers)	Local and state health departments	State (system leadership)	Program developers	University medical center
**3**	**Inner context**	Organizations delivering the EBP	Organizations delivering the EBPs	Churches	Local child welfare agencies	Public sector mental health systems, organizations delivering the EBPs	Organizations delivering the EBPs	Organizations delivering the EBPs	Local child welfare agencies	Organizations delivering the EBP	State-run prison
**4**	**Capital exchanged**	Money, EBP expertise, institutional knowledge, training/coaching capacity, flow of eligible clients, social capital with program developer	Money, EBP expertise, institutional knowledge, training/coaching capacity, flow of eligible clients, social capital with program developer	Money, EBP expertise, training	Money, referrals, case-level client information, social norms, staff	Money	Social capital, involvement of sources of power, strategic alignment with existing infrastructure and resources	Data/information, money	Money, policies and procedures, required expectations, performance reviews (job security, opportunities for promotion)	EBP information, implementation data, social norms and sense of community, networking opportunities	Provider time, free access to experts, positive publicity for the university, tangible resources
**5**	**Impact on outer and inner contexts**	Outer and inner: provides structure for measuring, reporting, and providing the EBP	Outer and inner: provides structure for measuring and reporting clinical outcomes, and providing the selected EBPs	Outer and inner: provides care pathway to increase screening and retention in care	Outer and inner: provides a care pathway; Inner only: expands staff, provides structure for data management	Outer and inner: money flow from taxpayers to local jurisdictions	Outer and inner: synergy across state-wide policy, existing resources and organizations, infrastructure for sustainable training	Outer only: public health mission and ability to obtain financial resources; No direct benefit to inner	Outer and inner: bridging factor failure contributed to implementation failure in both inner and outer contexts	Outer only: see rationale. Inner only: can help secure contracts, provides outward legitimacy, information sharing across sites, may increase awareness around internal processes	Outer only: community impact, access to new sources of grant money and other research resources Inner only: generation of program data and information about the target population
**Form dimensions**											
**1a**	**Rationale**	Community- academic partnerships	Political	Public health concern	Public health concern, model requirement	Political, public health concern	Political, regulatory, and community need	Regulatory, public health concern	Existing system structure	Program developers’ needs including the desire to better track implementation across sites	Individual’s decision-making and action
**1b**	**Implementation strategy**	No	No	Yes	No	Yes	Not explicit	No	No	No	No
**1c**	**Regulatory context**	Enforceable	Enforceable, voluntary	Encouraged, voluntary	Encouraged	Mandatory, enforceable	Voluntary	Mandatory	Mandatory, enforced	Voluntary but required if you want to provide SafeCare	Voluntary, unenforced
**2a**	**Duration**	Varied by system	Short-term	Long-term	Long-term	Short-term	Both	Long-term	Long-term	Long-term	Long-term
**2b**	**Changes across implementation phases**	Yes	Yes	Maybe	Maybe	Ideally	Yes	No	Yes	If program developer changes requirements	If policies change
**2c**	**Supports**	Contracting person in service system, existing resources	Service system division, existing resources	NGO, existing and new resources	Training from model purveyors, Regional Behavioral Health Boards, money, existing and new resources	Legislation, existing and new resources	Implicit support from state government, high degree of collaboration, existing resources	Legislation, new resources	Existing resources rather than specific supports	Varies by site, some existing some new	Flexibility that comes with an academic position and the ability to print program materials
**3a**	**Multiple systems**	Yes	No	Yes	Yes	No	Yes	Yes	Yes	Yes	Yes
**3b**	**General or specific**	General	General	Specific	General	General	General to EBPs for ASD	General	General	General	General
**3c**	**Outcomes**	EBP adoption, implementation and sustainment, staff turnover	EBP sustainment, staff retention	EBP sustainment	EBP fidelity, penetration, service outcomes (esp. timeliness)	EBP adoption and sustainment, workforce capacity, downstream clinical improvements	EBP adoption, implementation and sustainment	Equity, reduced number of new infections, EBP sustainment	Forced partnership led to failed EBP implementation, discontinuation of trainings, discontinuation of sustainment plan	EBP sustainment, “fidelity to the implementation process”	Reduced recidivism, improved infant health and bonding, increased client engagement, sustainability, reduced legal risk for the prison

## Results

### Bridging factor dimensions: organized by functions and forms

To categorize our bridging factor dimensions, we drew upon the concepts of functions and forms, a distinction that was originally proposed in the complex health intervention literature [59–61]. We conceptualize *function bridging factor dimensions* as core characteristics that define the bridging factor and speak to its purpose as it relates to EBP implementation. We conceptualize *form bridging factor dimensions* as characteristics that describe the specific structures, activities, and strategies that illustrate why and how the bridging factor has been customized to a local implementation experience. We envision that function dimensions will help researchers and practitioners identify the presence and purpose of a bridging factor, while the form dimensions will help researchers and practitioners understand how the bridging factor may be purposefully designed or modified to support EBP implementation in a specific context. We propose five function bridging factor dimensions and three form bridging factor dimensions. Each form dimension has three sub-categories. The dimensions are described below and summarized in Table 2.

**Table 2:** Bridging factor dimensions

**Function dimensions: core characteristics that define the bridging factor and speak to its purpose as it relates to EBP implementation**	
1. Type(s)	Relational tie, formal arrangement, and/or process?
2. Outer context	What is the outer context?
3. Inner context	What is the inner context?
4. Capital exchanged	What capital (e.g., fiscal, knowledge, norms, communication, resources) is exchanged through the bridging factor?
5. Impact on outer and/or inner context	How does the bridging factor impact the outer and/or inner context?
**Form dimensions: characteristics that help us understand the specific structures, activities, and strategies that illustrate why and how the bridging factor has been customized to a local implementation experience**	
1. Origin	
a. Rationale	Why and/or how was the bridging factor created?
b. Implementation strategy	Is the bridging factor a planned and deliberate implementation strategy?
c. Regulatory context	Is the bridging factor enforceable, mandatory, encouraged, and/or voluntary?
2. Dynamism	
a. Duration	Is the bridging factor short or long term?
b. Change across the implementation phases	How does the bridging factor change or require modifications across the implementation phases?
c. Supports	What resources or structures support the bridging factor and how stable are those supports? Does the bridging factor leverage existing resources and supports or require building new ones?
3. Scope	
a. Multiple systems	Does the bridging factor cross multiple service systems?
b. General or specific	Is the bridging factor general across EBPs or specific to a particular EBP?
c. Outcomes	How does the bridging factor affect implementation, clinical, and/or service outcomes?

### Five function bridging factor dimensions

1. Type (relational tie, formal arrangement, and/or process) [6]
2. Outer context
3. Inner context
4. Capital exchanged across the boundary of the defined outer and inner contexts
5. Impact the bridging factor has on the outer and/or inner contexts

The first dimension is specification of bridging factor type and we expect new types to emerge in future research. The next two function dimensions are identification of the outer and inner contexts, which is essential for defining what the “bridge” consists of. Our case studies reinforced the nested nature of implementation contexts (e.g.,  counties within states) and the importance of explicitly defining a project’s contextual boundaries. For the fourth dimension (capital exchanged) we take a broad view of the human, social, fiscal, knowledge, and time-related capital that may be relevant across the implementation phases. Examples include funding, EBP expertise and knowledge, client referrals, training and coaching capacity, social norms, program data and client information, and communication between individuals. For the fifth dimension (impact on outer and/or inner coreinforced the nested nature of implementation contexts

ntext), we assert that bridging factors can positively and/or negatively influence EBP implementation and sustainment. Case 8, for example, illustrates how a bridging factor can negatively affect implementation in the outer and inner contexts. Furthermore, because bridging factors span both contexts, we expect to see top-down (outer influencing inner) and bottom-up (inner influencing outer) processes. This bi-directional influence is reflected in Table 1, Function Row 5 which describes the impact that a bridging factor can have on outer and inner contexts. Our cases revealed that some bridging factors impacted both contexts in a similar way (e.g., cases that denote the same impact on outer and inner). However, bridging factors may influence outer and inner contexts differently (cases 9, 10) or potentially only show a tangible impact in either the outer or inner context (case 7). We acknowledge the limitations of retrospectively describing impact and expect the bi-directional influence and interaction that occurs through bridging factors to be more clearly described in future studies that prospectively and systematically examine top-down and bottom-up bridging processes.

### Three form bridging factor dimensions

1. Origin
  - Rationale
  - Implementation strategy
  - Regulatory context

The origin dimension helps researchers and practitioners understand the source of the bridging factor. The sub-categories are (a) rationale for the creation of the bridging factor, (b) the degree to which the bridging factor is a planned and deliberate implementation strategy (e.g., changing service provision contracts to require EBP implementation), and (c) regulatory context—that is, whether the bridging factor is enforceable (cases 1, 2, 5, 8), mandatory (cases 5, 7, 8), encouraged (cases 3, 4), and/or voluntary (cases 2, 3, 6, 9, 10). The origin dimension helps illustrate aspects of the bridging factor that are modifiable or fixed, and which stakeholders may need to be engaged as the bridging factor is developed or modified.

2. Dynamism
  - Duration
  - Change across the implementation phases
  - Supports

This dimension describes the dynamic or adaptive nature of the bridging factor and has three sub-categories. First (a) is the duration of the bridging factor. Knowing if the bridging factor is short- or long-term can help researchers and practitioners assess when it may influence EBP implementation, or at what phase the bridging factor will have the most influence. What constitutes a short- and long-term bridging factor is dependent on the implementation context and specific bridging factor. Second (b) is how the bridging factor changes or is expected to change across the implementation phases. Describing if and how the bridging factor changes (e.g., for political, regulatory, societal, or policy reasons) can bring to light environmental opportunities and constraints that affect the degree to which the bridging factor can be leveraged as a formal implementation strategy. Third, (c) bridging factor supports articulates two specific elements: needed resources or structures that support the bridging factor and whether or not the bridging factor leverages existing or requires new resources and supports. Bridging factor supports may include financial investment, contract requirements, or legislative mandates. We emphasize that these supports are directly related to the bridging factor and not supports for more narrowly defined EBP implementation activities.

3. Scope
  - Multiple systems
  - General or specific
  - Outcomes

This dimension describes the scope of the bridging factor. The first sub-category (a) is whether the bridging factor links multiple service systems such as child welfare and mental health or federal, state, provincial, and regional health systems. The second sub-category (b) is whether the bridging factor is general across EBPs or specific to a particular EBP (case 3). These sub-categories inform the breadth and depth of the bridging factor and have implications for the types of stakeholders needed to effectively link within and across systems and in service of one or multiple EBPs. This is important given that bridging factors often involve key partnerships and leveraging resources, processes, and arrangements that require stakeholder involvement in their creation or maintenance (even if the bridging factor is mandatory). The last sub-category (c) captures the impact (actual, potential, or expected) that the bridging factor has or is intended to have on outcomes.

In summary, our cases illustrate how contracting arrangements, fiscal incentives, partnerships, earmarked taxes, interagency collaborations, data sharing and accreditation processes, and even individuals can act as bridging factors that link an outer and inner context during EBP adoption, implementation, and sustainment. Recognizing the diversity of bridging factors (in our cases as well as those yet to be identified), we offer function and form dimensions as a way to organize and report information across implementation studies, with the hopes of building a bridging factors knowledge base. Function dimensions include specifying the type of bridging factor, describing the outer and inner contexts (the contextual boundaries of the bridge), capital exchanged, and impact on outer and inner contexts. The form dimensions help us understand what a bridging factor looks like in a specific implementation setting. These dimensions are origin, dynamism, and scope (with various sub-forms in each). We next outline our bridging factors research agenda.

## Recommendations for a bridging factor research agenda

### For theory

There are numerous ways that the bridging factors construct can be utilized in implementation theories, frameworks, and models. Bridging factors provide a way to more deeply consider how outer and inner contexts are related and what strategies might be used to bridge them. In turn, those approaches might generalize to other public health concerns, diseases, or clinical and service settings. Considering how bridging factors operate similarly or differently across levels and in various settings (e.g., countries, provinces, states, counties, etc.) combined with frameworks such as EPIS, CFIR, ISF, and others can help us develop ways to integrate, understand, and assess the differential impact and tailoring needs of bridging strategies for specific contexts. For example, if we narrow consideration to setting and clinical concern (e.g., national health system and addressing depression) there might be a number of multilevel mechanisms by which the outer context can influence clinical practice through a planned implementation strategy (e.g., medication formularies, funding for psychosocial interventions). This may vary by context, for example, in LMICs where mental health may be a lower priority than other public health concerns.

It may also be helpful to consider bridging factors within the major types of frameworks categorized by Nilsen [62]. As noted by Nilsen, determinant frameworks may be limited in regard to the process of conducting implementation [62]. To address this gap, determinant frameworks might be adapted to consider bridging factors.Process models may be tailored and/or adapted to consider how bridging factors influence key processes, including the critical multilevel mechanisms that allow strategies at one level to influence implementation outcomes at another level [63]. Finally, evaluation frameworks may benefit from actively including the assessment of bridging factors as they may support or hinder an implementation effort. Regardless of the category of model, theory, or framework, we recommend considering and integrating bridging factors.

### For measurement and design

Bridging factors add another element to consider in research design and measurement. It could be argued that without due consideration of bridging factors, studies do not portray or assess the full picture of implementation, which may result in Type 3 errors. This defies the principles of implementation science, and may inadvertently lead to inappropriate data analyses if the bi-directional and multilevel nature of implementation influences are not considered. The multilevel nature of bridging factors needs to be accounted for in any analysis that involves these cross-level linkages. Some bridging factors may be captured using established quantitative scales (e.g., leadership). However, a recent review of measures of outer context constructs used in behavioral health research highlighted the limited quantitative investigation of system-level and policy influences both in terms of quantity and quality [64]. Interestingly, 7 of the 20 measures found in this review were related to cosmopolitanism, which is defined in CFIR as the “degree to which an organization is networked with other external organizations,” and thus links to the concept of bridging factors. Unfortunately, the quality of the measures was poor with the highest psychometric score being 5 out of possible 36. Similar reviews have also been conducted in public health and community settings [64] and health care quality improvement initiatives [65].

Nevertheless, we consider qualitative methods as being key to measuring and understanding bridging factors. Qualitative methods are particularly well suited to assess complexity and context and may be included in mixed methods studies. Qualitative methods (e.g., interviews and focus groups) may elicit bridging factors that were not considered in the study development or factors that were not present in the early phases of the implementation process. Qualitative methods are also able to capture the dynamic nature of bridging factors and the varying degree of influence that they have throughout the implementation process from different outer and inner stakeholder perspectives. We recommend that implementation researchers consider pursuing funding for in-depth, semi-structured interviews and/or focus groups to describe and explain the influence of bridging factors on implementation strategies, processes, and outcomes. The questions posed in Table 2 can be a foundation for interview scripts.

### For reporting

As our cases suggest, bridging factors can influence the implementation process, and they may facilitate or hinder activities within each phase of implementation. They may also interact with implementation strategies and illuminate the mechanisms integrated within these efforts. For example, case 6 underscored the importance of the capital exchanged to facilitate the preparation, implementation, and sustainment of the California Professional Training and Information Network (CAPTAIN) [66]. This included leveraging social capital through the involvement of key stakeholders spanning multiple levels and the incorporation and alignment of existing infrastructure and resources. However and similar to the history of implementation strategies and mechanisms [34, 67], there has been limited reporting and specifying of bridging factors to date, likely due to limited awareness and development of guidance around this key group of implementation factors. We recommend that further specifying and reporting of such factors is needed to continue advancing the field and extending the impact of implementation efforts. This includes explicit identification, provision of detailed descriptions, and measurement of their impact [30].

Similar to the rationale for further specification of other implementation methods, bridging factor specification will help  efforts to replicate or scale-up, especially given their potential as key determinants. Efforts to replicate and/or scale-up the CAPTAIN model statewide, for example, would be greatly impeded if the alignment between existing outer context priorities and policies and involvement of existing inner context resources had not been explicitly specified and reported, permitting outer-inner context integration in further implementation efforts [68]. In addition to the proposed dimensions, the growing set of guidelines and recommendations for specifying and reporting implementation methods (e.g., use of implementation strategies and implementation frameworks [67, 69]) aligns well with this recommendation.

### For outcomes research

Because bridging factors bi-directionally influence outer and inner contexts, they may influence implementation outcomes across contexts. For example, our partnership case studies illustrated how bridging factors can influence implementation outcomes in both outer and inner contexts. Partnerships developed from shared goals, trust, and high levels of engagement among outer and inner context stakeholders may positively impact levels of sustainment, fidelity, and reach in LMIC faith-based organizations (case 3), child welfare (case 4), and autism service settings (case 6). Case 8 demonstrated how the dissolution of a forced (e.g., legislatively or structurally mandated) partnership helped to explain why an EBP was discontinued despite the presence of successful implementation and clinical outcomes.

As the field of implementation science evolves, and with the hope that bridging factor conceptualization continues to be refined, it will be important for researchers to systematically and rigorously test the impact of bridging factors on implementation outcomes and the contextual considerations that can explain this impact. For example, the question of under which conditions certain bridging factors positively or negatively impact implementation outcomes is ripe for future investigation. We suggest using Proctor et al.’s taxonomy as a starting point to evaluate the impact of bridging factors [70].

### For research about implementation phases and stages

In addition to their role in supporting key cross-context linkages between the outer and inner contexts, bridging factors are also a cross-cutting feature across implementation phases and stages. In practice, the impact of a bridging factor may only be felt in one or two phases or stages of implementation. Bridging factors may be specifically designed to be short-term or time limited. However, there is immense potential for these factors, especially those designated as long-term, to exert their impact across all phases or stages of implementation. Bridging factors can also impact the process and progress within an implementation stage, serving to accelerate or impede the completion of implementation activities within each stage and the number of stages completed. For example, case 2 highlighted how the formal oversight structure and supports that accompanied the fiscal incentives during the preparation phase facilitated large-scale EBP implementation and sustainment countywide [71]. Given the dynamic and evolving nature of bridging factors, modifications may be made to accommodate the varying foci or goals of each implementation phase or stage, as well as be a response to the evolving demands of the outer and/or inner contexts.

### For practice and policy

Well-specified bridging factors have great potential to serve as policy levers that can inform and change practice. A clear understanding of the roles of bridging factors and their impacts on outer and inner context variables, including implementation outcomes, can help guide decisions about policies that inform EBP implementation and practice delivery. For example, case 5 suggested that the structure of a policy (taxes) has meaningful implications on decisions for service delivery (e.g., types of services, interventions selected, workforce capacity to deliver services). Similarly, case 2 illustrated the importance of a solid and stable infrastructure to support the execution and ongoing maintenance of a system-wide transformation of care delivery. As described in earlier sections, it is critical for bridging factors to be well specified and defined.

### Challenges

We found that some bridging factors dimensions were easier to identify and report (e.g., if it is a planned implementation strategy) than others (e.g., capital exchanged). Additionally, in-depth system knowledge was necessary to understand how bridging factors operated and influenced implementation processes. This required collecting data from individuals who had rich experience with a particular setting. Some information may also be sensitive or of a political nature and therefore more difficult to gather and use. Furthermore, we found that the nested nature of the outer and inner contexts can make it difficult to define the boundaries of a particular bridging factor. It is also likely that there are similar processes that bridge levels within outer and inner contexts. That being said, we encourage researchers to be as specific as possible when identifying the outer and inner contexts of interest. A final challenge of conducting bridging factors research  is that operationalization is still in its infancy. As a result, existing literature does not explicitly report and measure bridging factors and we had to rely on our research experiences and discussions. We hope that this manuscript offers initial guidance for reporting and measuring bridging factors in future work.

## Conclusions

In implementation science, bridging factors research is in its early stages. Previous work formally added the construct to the EPIS framework and illustrated a specific methodological approach and contracting example [5, 12]. This paper recognizes that the next steps are to report and measure bridging factors so that they can be systematically tested and proactively leveraged during EBP implementation and sustainment. Our primary task for this paper was to make bridging factors more concrete and specific. To achieve this, we examined diverse bridging factor cases and articulated dimensions that can help to describe bridging factors functions and forms. Finally, we put forth recommendations for theory, measurement and design, reporting, outcomes, and research that spans the implementation phases and stages. We also began a discussion of the practice and policy implications of this work. We hope that this paper inspires future bridging factors research so that we can strengthen linkages across outer and inner contexts, improve implementation outcomes, and enhance the public health impact of EBPs.

## Supplementary Information

**Additional file 1.**

## Data Availability

Not applicable.

## Abbreviations

EBPs: Evidence-based practice
EPIS: Exploration, Preparation, Implementation, and Sustainment
CFIR: Consolidated Framework for Implementation Research
RE-AIM: Reach Effectiveness-Adoption Implementation Maintenance
ISF: Interactive Systems Framework
LMIC: Low- and middle-income country
