# Supplementary material for: Forms and functions of bridging factors: specifying the dynamic links between outer and inner contexts during implementation and sustainment
Source: Implement Sci. 2021 Apr 1;16:34. doi: 10.1186/s13012-021-01099-y (PMC8015179; doi:10.1186/s13012-021-01099-y)
Supplement: Supplementary file 1 — Additional file 1. [file 13012_2021_1099_MOESM1_ESM.docx]

Additional file 1. Bridging factor case studies.

**Case 1: Contracting arrangements**

This case describes how contracts are a formal arrangement (function 1) type of bridging factor. The evidence-based practice (EBP) is SafeCare®, a highly prescriptive, modular, flexible, and well-researched intervention designed to reduce and prevent child maltreatment. The outer context (function 2) is public sector child welfare systems while the inner context (function 3) is community-based organizations that are implementing SafeCare. Capital exchanged (function 4) between the public sector child welfare systems and community-based organizations through contracting arrangements included money, EBP expertise, institutional knowledge, training and coaching capacity, flow of eligible SafeCare clients, and social capital with program developers at the National SafeCare Training and Research Center. This bridging factor impacted both the outer and inner contexts (function 5) in that the contract arrangements created structure for providing SafeCare along with ongoing training, fidelity monitoring and coaching.

Community-academic partnerships influenced the early development of these SafeCare contracting arrangements (form 1a). The contracting arrangements were not designed to be a planned and deliberate implementation strategy (form 1b), but they were part of a larger implementation strategy that included contracting with and accountability to the public sector child welfare system (form 1c). The duration (form 2a) of the contracting arrangements varied by child welfare system. The contract content, statements of work, and specifications evolved over the course of SafeCare implementation (form 2b). Supports (form 2c) for the contracting arrangements included a contracting person or team (each child welfare system had its own arrangement) and existing administrative resources in the outer and inner contexts.

In some instances, the contracting arrangements involved other systems besides child welfare (form 3a). Overall contract design and format was applicable across EBPs other than SafeCare (form 3b). As a bridging factor, contracting arrangements positively influenced SafeCare adoption, implementation, and sustainment (form 3c). They also helped to mitigate staff turnover in the implementing organizations (form 3c). Previously published work provides more details about this case [1].

**Case 2: Policy-driven fiscal incentive**

This case describes how a policy-driven fiscal incentive to implement EBPs is a formal arrangement (function 1) type of bridging factor. The context is a transformation of mental health service delivery in the Los Angeles County Department of Mental Health (outer context, function 2) through the Prevention and Early Intervention (PEI) Plan approved by the Mental Health Services Oversight and Accountability Commission in 2009. The PEI Plan provided a fiscal incentive for public sector clinic and school-based mental health organizations (inner context, function 3) to obtain reimbursement for implementation of selected EBPs. Provider EBP training and implementation support (e.g., technical assistance) was first made available for an initial six EBPs to address a range of child mental health needs. The PEI Plan also impacted adult mental health services but we only focus on the child-focused services in this case. The EBPs included (form 3b) Cognitive Behavioral Interventions for Trauma in Schools, Child-Parent Psychotherapy, Managing and Adapting Practices, Seeking Safety, Trauma-Focused Cognitive Behavior Therapy, and Triple P Positive Parenting Program.

Capital exchanged (function 4) between Los Angeles County Department of Mental Health and the implementing mental health organizations through the PEI Plan included: 1) money, 2) establishing contracts with the EBP developers for training, consultation and certification, 3) technical expertise to amend the billing system for submitting reimbursement to accommodate delivery of these EBPs, 4) and staffing changes to support the ongoing training and monitoring of EBP delivery. This bridging factor impacted the outer and inner contexts (function 5) in that the PEI Plan created structure for measuring outcomes, reporting, and providing the selected EBPs within organizations.

Public concern about the quality and types of mental health service needs and fiscal policy changes influenced the early development and ongoing evolution of the PEI Plan (form 1a). Although not explicitly designed to be an implementation strategy, the nature of the fiscal incentive to deliver EBPs offered the opportunity for EBP implementation to be prioritized in Los Angeles County mental health service delivery (form 1b). The PEI Plan was enforced by Los Angeles County Department of Mental Health through an oversight structure (form 1c). The duration and specifications (form 2a) of the PEI Plan evolved over time but required regular reporting for re-approval (form 2b). Supports (form 2c) for the PEI Plan included initial implementation support for provider training, initial technical assistance site visits, assignment of Practice Leads to oversee compliance, and Los Angeles County coordination of ongoing provider training and monitoring of training completion and certification (form 3a).

The overall design of the PEI Plan regarding implementation and monitoring was applicable across the selected EBPs but there were EBP-specific aspects including the training requirements, fidelity assessment and clinical outcomes used (form 3b). As a bridging factor, this fiscal incentive through the PEI Plan has been associated with increased rates of reach, sustainment of many of the initial EBPs and expansion to new EBP implementation (form 3c) [2]. A study protocol provides more details about the context for the case and example implementation research to understand its multi-level impact [3].

**Case 3: Community-academic partnerships in a LMIC**

This case describes how community-academic partnerships in a low-and-middle-income country (LMIC) implementation are a relational (function 1) type of bridging factor. The EBP is the Health Beginnings Initiative, a culturally tailored, family-centered and congregation-based intervention designed to prevent mother-to-child transmission of HIV through HIV testing and care linkage (form 3b). The Healthy Beginnings Initiative provides free, integrated on-site health screens, including HIV testing, during a baby shower as part of church activities, with subsequent linkage to follow-up care as needed. The outer context (function 2) is the funding agencies of the Healthy Beginnings Initiative including the US National Institutes of Health and the Nigerian Ministry of Health that provides the funding and structure for medical care while the inner context (function 3) is individual churches where the Healthy Beginnings Initiative was implemented.

Two types of community-academic partnerships were forged to support this work. The first was a partnership with the network of churches in Enugu State in southeast Nigeria. Church attendance is over 90% in Enugu and the HIV seroprevalence rate was greater than the national average. Leveraging the community-based infrastructure of churches that were viewed as a trusted and commonly accessed organization was a culturally relevant entry point for HIV testing implementation. The second partnership was through a local non-governmental organization (NGO), the Healthy Sunrise Foundation, that worked with a local intermediary, Prevention, Education, Treatment, Training and Research-Global Solutions of Nigeria, to offer training in the Healthy Beginnings Initiative to study staff, church-based volunteer health advisers and priests. Capital exchanged (function 4) between the outer and inner contexts through the two partnerships included money, EBP and HIV expertise, institutional knowledge, training and coaching capacity and flow of eligible pregnant women to antenatal care. This bridging factor impacted both the outer and inner contexts, but primarily the inner (function 5) in that the partnerships created new pathways and structures for providing HIV testing and care linkage.

Policy calls, including the WHO and the US President's Emergency Plan for AIDS Relief (PEPFAR), coupled with the high prevalence of HIV in LMICs and evidence that HIV testing uptake with traditional clinic-based approaches was limited in LMICs shaped the need for disruptive, community-based approaches to maximize uptake and sustainment of HIV testing and care linkage (form 1a). The partnership with the Sunrise Foundation originated, in part, because the funding agencies for the Healthy Beginnings Initiative trial mandated inclusion of a local PEPFAR-supported partner with relevant health delivery experience (form 1a).

The partnerships were designed to be a planned and deliberate implementation strategy for the Healthy Beginnings Initiative (form 1b), but they were not necessarily enforceable since this was in the context of a research trial (form 1c). The duration (form 2a) of the partnerships was linked to the funding arrangements of the Healthy Beginnings Initiative and the partnerships have continued with funding support from the NIH, PEPFAR and the Healthy Sunrise Foundation (form 2b). Supports (form 2c) for the partnerships included leveraging existing training resources and infrastructure from the local NGOs to provide training and ongoing support to the churches and volunteer health advisers implementing the intervention (form 3a). The partnerships were specifically designed for the church infrastructure and implementation of the Healthy Beginnings Initiative (form 3b). As a bridging factor, the partnerships successfully supported implementation of the Healthy Beginnings Initiative, which resulted in high rates of reach, fidelity and HIV testing uptake (form 3c). The outcomes study offers greater details about this case [4].

**Case 4: START model partnerships**

This case describes how partnerships are a relational tie (function 1) type of bridging factor. The EBP is STARecoveryTeams (START), a nationally implemented child welfare model designed to provide brief screening followed by coordinated linkages to effective substance use treatment. The outer context (function 2) is substance use treatment organizations in the community, while the inner context (function 3) is the local, public serving child welfare agencies. These partnerships can be informal or formally codified in a memorandum of understanding (MOU) or contract. The capital exchanged (function 4) between the outer and inner contexts is money (in the form of formal contracts), referrals, case-level client participation and outcome information, social norms, and staff (as the partnership is a way to expand staff without having to hire).

The bridging factor impacts outer and inner contexts (function 5) in several ways. Many of the child welfare agencies contract with the substance use treatment organizations for the Family Peer Mentor position. This role requires specialized expertise and expanding workforce capacity.

Additionally, substance use treatment organizations agree to provide treatment to caregivers. This can be an informal referral or a formal MOU where the child welfare agency agrees to waive waitlist requirements for the parent needing treatment. Finally, there is joint case planning and information sharing across the inner and outer contexts as a result of this bridging factor. The child welfare agencies want Family Peer Mentors and substance use treatment providers to participate in family team meetings with the child welfare caseworkers to determine case goals. Meeting participants share information about client needs, “compliance,” and progress.

START was designed based on public health concern that an increasing number of children are in foster care due to the opioid crisis. Therefore, partnerships between the child welfare system and substance use treatment providers is essential (form 1a). Partnerships formed to implement START are unique in that it is a deliberate requirement for the model (form 1b). Treatment developers highly encourage agencies to have a formal partnership codified in an MOU or contract if necessary (form 1c). The intended duration (form 2a) of the partnerships formed is long-term, but whether this eventuates remains to be seen and is under investigation. Similarly, if the partnerships change over the phases of implementation is not yet known (form 2b).

Supports (form 2c) for the bridging factor included training and technical assistance from START purveyors, and money (child welfare agencies receive money through a contract or subcontract and many of them turn it around and use it to “buy” a Family Peer Mentor or specialized services from the substance use treatment organizations). Third party coordinating bodies in the community (e.g. Regional Behavioral Health Boards, local service coalitions, or the courts) have the potential to support bridging by identifying and introducing partners. This bridging factor involves collaborative strategies across multiple systems (form 3a) and are general (form 3b) in that they could be applied to other cross-system EBPs. It is hoped that the partnerships formed as part of the implementation of START will positively influence START fidelity, penetration and service outcomes, e.g., timeliness (form 3c). There is a published study protocol for this case [5].

**Case 5: Earmarked taxes**

This case describes how earmarked taxes are an example of a formal arrangement (function 1) type of bridging factor. Earmarked taxes—defined as taxes for which revenue can only be spent on specific activities—for mental health services are an increasingly popular legislative policy in jurisdictions across the United States (e.g., California, Washington State, Missouri, Illinois, and Colorado). The ways in which the earmarked revenue can be spent varies across jurisdictions. For example, insights from a published policy column [6] contrasted two statewide earmarked mental health taxes that exist in California and Washington, and only the former includes opportunities to restrict funding for specific EBPs or promising practices. Regardless of the specific legislation, there are shared aspects that unite the earmarked mental health tax policies as a bridging factor exemplar. The outer context (function 2) is the state-level sociopolitical context (e.g., state legislators, voting constituents) that authorizes taxes. The inner context (function 3) is both the public sector mental health systems that receive and distribute the earmarked dollars and the mental health service delivery organizations that are responsible for providing mental health services. Money via tax revenue is the primary capital exchanged (function 4) between the authorizing legislation, the mental health systems and mental health service delivery organizations. This bridging factor impacts the outer and inner contexts (function 5) in that the taxes create a structure for measuring, reporting, and providing mental health services as well as fiscal oversight of tax spending.

A variety of influences shaped the initiation and trend of earmarked mental health taxes. These included mounting public concern about behavioral health, demand for policies that increase access to care, and constituents’ willingness to pay higher taxes for behavioral health services [7, 8]. Concern, demand and willingness were also coupled with insights from implementation science that behavioral health EBPs exist but require implementation support, particularly sufficient funding, to maximize success and sustainment (form 1a). Although not deliberate implementation strategies, the taxes align with several outer context implementation strategies (form 1b) such as providing access to new funding, changing incentive structures, and mandating the use of EBPs but they hinge on the successful design and implementation of the tax itself.

Nonetheless, the taxes were enforceable and enforced by authorizing legislation and oversight structures with which mental health systems comply (form 1c).

The duration (form 2a) of the taxes varies by jurisdiction and requires ongoing re-authorization. For California and Washington, their respective legislation has been continuously approved since 2005 (form 2b). Supports (form 2c) for the taxes included dedicated funds for behavioral health service delivery (wide variability in how this was operationalized across jurisdictions) that allowed for leveraging, expanding and building new resources and programs. In the case of Washington, the tax structure involved multiple systems that provide care for individuals with mental health and substance use disorders (form 3a). Because EBP delivery is not universal or mandated, the overall tax design of implementing jurisdictions appears flexible across mental health service options (form 3b). The impact of earmarked mental health taxes as a bridging factor are not well known. However, emerging evidence suggests that the taxes are associated with both positive (e.g., increased reach, service utilization and symptom improvement) and negative effects (e.g., increased provider stress) (form 3c). Richer examination of earmarked taxes as a bridging factor in California and Washington will be explored in a newly funded NIMH R21 (MH125261).

**Case 6: Statewide interagency collaboration**

This case describes a statewide interagency collaboration, entitled the California Autism Professional Training and Information Network (CAPTAIN), which aims to facilitate the scale-up of EBPs for individuals with autism spectrum disorder (ASD) across education and developmental disabilities service sectors. CAPTAIN is a relational tie (function 1) type of bridging factor. Legislation and statewide initiatives prioritizing the implementation of EBPs for individuals with ASD served as the impetus for the formation of this partnership. Specifically, the creation of an interagency, collaborative professional development and provider training curriculum for ASD was identified as a primary method to increase adoption of ASD EBPs, prompting the development of CAPTAIN.

CAPTAIN includes a collaborative leadership team and a large number of members representing multiple educational and community agencies throughout the state of California. CAPTAIN members provide training and coaching in ASD EBPs and continue to meet regularly as regional teams. The outer context (function 2) is statewide policy and legislation regarding ASD service provision and the inner context (function 3) comprises organizations and providers delivering ASD EBPs. Capital exchanged (function 4) includes social capital, involvement of key stakeholders and/or decision makers spanning multiple levels (e.g., policy-makers, system and agency leaders, ASD experts), and alignment with existing infrastructure and resources. CAPTAIN impacts the outer and inner contexts (function 5) by enhancing provider training and subsequent EBP implementation (inner context), as well as helping to inform policy for ASD best practice recommendations (outer context).

The increased prioritization of improving ASD services, including the goal of establishing a professional training model, and community needs influenced the establishment of CAPTAIN (form 1a). The creation of this partnership was not explicitly designed to be an implementation strategy (form 1b) and participation in CAPTAIN is voluntary (form 1c). The duration of CAPTAIN is long-term (form 2a), with evolving changes and modifications across implementation phases (form 2b). For example, funding for CAPTAIN and CAPTAIN activities was initially very limited; however, existing resources and in-kind support for leader participation and registration from initial training summits were leveraged to cover the cost of activities. These resources, as well as additional funding obtained, support the sustainment of CAPTAIN (form 2c). In terms of scope, CAPTAIN includes multiple relevant agencies providing services for individuals with ASD (form 3a) and pertains to a broad range of ASD EBPs versus one specific EBP (form 3b). CAPTAIN directly influences the adoption, implementation and sustainment of ASD EBPs (form 3c). A recently published paper provides more details regarding the formation and sustainment of CAPTAIN [9].

**Case 7: Data sharing process**

This case describes a process (function 1) type of bridging factor. This bridging factor is a data sharing, monitoring, and feedback process that supports evidence-based HIV interventions. The interventions involved in this bridging factor are part of the Ending HIV Epidemic Initiative, a national plan to end the HIV epidemic in the United States by 2030. The outer context (function 2) is comprised of city and state health departments that are expected to adhere to guidelines put forth by the Centers for Disease Control and Prevention. The inner context (function 3) is community-based organizations, AIDS service organizations, and primary care clinics that implement the interventions and submit data. Capital exchanged (function 4) between the health departments and organizations/clinics through this process are data and information, which is then used by the health departments to make decisions about where to allocate money. In this case, the bridging factor did not provide any direct benefit to the inner context. The bridging factor was, however, imperative to the public health mission of the outer context. It also enhanced the outer context’s ability to obtain financial resources in that the data are used at the state and federal levels to determine resource allocation in the form of new funding opportunity announcements to support HIV interventions in jurisdictions with increasing infection rates (function 5).

The public health goals associated with the Ending HIV Epidemic Initiative and associated regulations motivated the development of this bridging factor (form 1a). The data sharing, monitoring, and feedback process was not designed to be a planned and deliberate implementation strategy (form 1b), but it is mandated at the county level (form 1c). This bridging factor is long-term, consistent, and institutionalized (form 2a). The process could change for political reasons, if regulations shift, or if there are advances in the way that HIV transmission is traced (form 2b). Initiating this bridging factor required building new information technology infrastructure and health information technology functionality (form 2c).

This bridging factor crosses multiple systems—including primary care settings and hospitals, local, state, and federal departments of public health, and the criminal justice system (form 3a)—and it is applicable across evidence-based HIV interventions (form 3b). As a bridging factor, this process supports equity (in terms of allocating resources to service deliverers in jurisdictions with greater need) and reduced number of new infections. It also supports the sustainment of evidence-based HIV interventions because it is a way to provide sustained funding and ensure that the limited amount of available funding is distributed to the areas of greatest need (form 3c). This bridging factor draws from work funded by P30 DA027828 and R01 MH118213.

**Case 8: Partnership between state and local child welfare agencies**

This case is an example of a bridging factor that harmed EBP implementation efforts. The bridging factor is a partnership, which falls within the relational tie (function 1) type of bridging factor. The EBP is R^3^, a supervision-focused implementation approach [10]. The outer context (function 2) was a State (system leadership) in the United States of America and the inner context (function 3) was local child welfare agencies. Capital exchanged (function 4) between the State and the local child welfare agencies through this partnership were policies and procedures (e.g. weekly implementation calls between State leadership, regional leadership, and R^3^ developers were scheduled prior to partnership failure to discuss structuring regions for trainings, coaching, and monitoring), required expectations and performance reviews (which led to job security and opportunities for promotion), and money. The bridging factor also reinforced the hierarchy between the State and local child welfare agencies, including regional leadership and frontline staff. This partnership aimed to facilitate the statewide adoption of R^3^. When the partnership failed, R^3^ sustainment failed in both the inner and outer contexts (function 5).

The partnership between the State and local child welfare agencies existed before R^3^ implementation (form 1a). The partnership was a system structure that acted as the backdrop for R^3^ implementation and was not a planned strategy that was specifically created or used to support R^3^ implementation (form 1b). Additionally, this partnership was mandatory, enforced (form 1c) and long-term (form 2a) in that it should last throughout the full implementation process—from Engagement to Sustainment. Administrative changes occurred within the partnership for political reasons (form 2b). More specifically, a new elected official put new people in place who had different priorities that conflicted with R^3^ implementation. It was described as a “forced partnership” and specific supports were not provided (form 2c). The partnership crossed multiple child and family public serving systems including child welfare, juvenile justice, and self-sufficiency (form 3a), and again, was not specific to the R^3^ intervention (form 3b). Outcomes impacted by this bridging factor include failed implementation, discontinuation of R^3^ trainings, and discontinuation of the sustainment plan (form 3c).

**Case 9: Site-level accreditation process**

This case describes how site-level accreditation is a process (function 1) type of bridging factor. The EBP is SafeCare®, a modular, well-researched and flexible intervention designed to reduce and prevent child maltreatment. The outer context (function 2) is the SafeCare program developers (National SafeCare Training and Research Center, NSTRC) while the inner context (function 3) comprises community-based organizations that are implementing SafeCare. Capital exchanged (function 4) between NSTRC and community-based organizations through the site-level accreditation process includes information about the intervention, implementation data (e.g. the number of providers delivering SafeCare, client demographics, fidelity data), and relational capital built through networking opportunities with NSTRC and other implementing sites. Through accreditation activities (e.g. group phone calls) this process also creates social norms around the intervention and a sense of community among providers.

This bridging factor impacted the outer context (function 5) in a variety of ways. Site-level accreditation allows NSTRC to better track SafeCare implementation across sites and creates a more streamlined approach to disseminating information. Regular monitoring of implementation quality (through the accreditation process) also allows NSTRC to protect the “SafeCare brand”. This bridging factor impacts the inner context (function 5) because it allows the community-based organizations to keep providing SafeCare with fidelity. Additionally, this bridging factor can help community-based organizations secure contracts and demonstrate SafeCare competency to funders and the broader community. In some cases, this bridging factor increased awareness around internal processes related to SafeCare implementation (e.g. standardizing the support provided to home visitors).

This bridging factor was formed by NSTRC, the program developer (form 1a). In addition to the information provided above, NSTRC wanted to use the accreditation process to build a network and collaborative community of SafeCare organizations and providers. The accreditation process was not designed to be a planned implementation strategy (form 1b), but it is required if the community-based organization wants to provide SafeCare. Achieving accreditation allows community-based organizations to maintain their SafeCare contracts (form 1c). This bridging factor is long-term (form 2a) and maintained through annual re-accreditation. NSTRC updates the accreditation process and requirements as needed (form 2b), and both NSTRC and the community-based organizations are required to contribute time and money to support accreditation requirements (form 2c). This bridging factor crosses multiple systems in that SafeCare implementation may involve multiple child serving systems and the accreditation process is utilized internationally (form 3a). Accreditation has been used by other program developers (form 3b), thus, accreditation processes are likely a generalizable bridging factor. In this case, accreditation as a bridging factor impacts EBP sustainment, and fidelity to the EBP delivery process (form 3c).

**Case 10: Individual as a bridging factor**

This case describes a person who acted as a bridging factor, while implementing programs for incarcerated pregnant and postpartum women. It is an example of a relational tie (function 1) type of bridging factor. The outer context (function 2) is a university medical center and the inner context, where the programs are being delivered (function 3), is a state-run prison. The individual acting as a bridging factor is a faculty member who goes to the prison and implements portions of the programs directly as a part of the prison’s volunteer program, as well as links individuals in her network to the state-run prison to support additional program components. The outer and inner contexts are connected in that the prison has a contract with the university medical center to provide pre- and post-partum care onsite and all of the incarcerated women from this prison deliver at the center.

Capital exchanged through the bridging factor (function 4) includes provider time (volunteer hours from the bridging factor and connection to a lactation consultant), access to experts (at no cost), positive publicity for the university medical center and prison, and tangible resources (e.g. large donations of supplies, medical-related information and posters for the prison). At times, positive publicity facilitated by the bridging factor’s involvement helped facilitate the donation of supplies for the program. The bridging factor impacted inner context goals through the generation of program data and information about the target population (function 5). The bridging factor impacted outer context goals in that the program aligns with their strategic plan to have community impact, and implementation of the program allows the university to access new sources of grant money and other resources for research because it is a unique implementation environment and specialty population (function 5).

This individual noted that she “didn't realize I was going to be a bridging factor" but did not know what would happen to program implementation if she did not lead implementation (form 1a). She also wanted to support prison leadership’s desire to provide more maternal care-focused programming. Essentially, the bridging factor formed because there was a strong community need coupled with a clear open window for action that fit with this individual’s values. This bridging factor was not a planned implementation strategy (form 1b) and is voluntary and unenforced (form 1c). However, taking on this bridging factor role is rewarded, e.g. the university recognizes innovative implementation research. This individual has acted as a bridging factor for multiple years (form 2a). The scope and intensity of involvement as a bridging factor changed across the implementation stages (form 2b), and could evolve if policies changed. One example would be if this role was a more formal part of the bridging factor’s job duties or if the medical contract could cover certain activities that the bridging factor currently does.

This particular bridging factor played the heaviest roles in the needs assessment (exploration/ preparation) phase and is also extremely important during the sustainment phase in terms of coordinating action across entities, catching when things are not happening that are supposed to, and ensuring that stakeholders continue to be in contact with one another. Two specific supports for this individual-level bridging factor are the flexibility that comes with an academic position and the ability to print program materials (form 2c). This bridging factor crosses multiple systems (form 3a) around the program implementation, including the university medical center, prison, and local non-profit community. This bridging factor is general across EBPs (form 3b) and outcomes impacted by this individual’s involvement as a bridging factor (form 3c) include reduced recidivism among program participants, improved infant health and bonding, increased client engagement in the program, program sustainability, and reduced legal risk for the prison.

Work on this case study was supported by a career development award funded by the National Institute on Drug Abuse (K23DA048162; PI: Zielinski). The project described was supported by the Translational Research Institute (TRI), grant UL1 TR003107. The content is solely the responsibility of the authors and does not necessarily represent the official views of the National Institutes of Health.

**Additional file 1 References**

1. Lengnick-Hall R, Willging C, Hurlburt M, Fenwick K, Aarons GA. Bridging factors linking outer and inner contexts: a longitudinal study of the role of contracting in implementation and sustainment. Implement Sci. 2020;15:43.
2. Brookman-Frazee L, Stadnick N, Roesch S, Regan J, Barnett M, Bando L, et al. Measuring sustainment of multiple practices fiscally mandated in children’s mental health services. Adm Policy Ment Health. 2016;43:1009-1022.
3. Lau A S, Brookman-Frazee, L. The 4KEEPS study: identifying predictors of sustainment of multiple practices fiscally mandated in children’s mental health services. Implement Sci. 2015;11:1.
4. Ezeanolue EE, Obiefune MC, Ezeanolue CO, Ehiri JE, Osuji A, Ogidi AG, et al. Effect of a congregation-based intervention on uptake of HIV testing and linkage to care in pregnant women in Nigeria (Baby Shower): a cluster randomised trial. Lancet Glob Health. 2015;3:e692-e700.
5. Bunger AC et al. Establishing cross-systems collaborations for implementation: protocol for a longitudinal mixed methods study. Implement Sci 2020;15:55.
6. Purtle J, Stadnick NA. Earmarked taxes as a policy strategy to increase funding for behavioral health services. Psychiatr Serv. 2020;71:100-104.
7. McGinty EE, Goldman HH, Pescosolido BA. Communicating about mental illness and violence: balancing stigma and increased support for services. J Health Polit Policy Law 2018;43:185–228
8. Stone EM, McGinty EE. Public willingness to pay to improve services for individuals with serious mental illness. Psychiatr Serv 2018; 69:938–941.
9. Suhrheinrich J, Schetter P, England A, Melgarejo M, Nahmias, AS, Dean M, et al. Statewide interagency collaboration to support evidence-based practice scale up: the California autism professional training and information network (CAPTAIN). Evid Based Pract Child Adolesc Ment Health. (in press).
10. Saldana L, Chamberlain P, Chapman J. A supervisor-targeted implementation approach to promote system change: the R3 model. Adm Policy Ment Health. 2016;43:879–892.
